# Shiga Toxin-Producing *Escherichia coli* Outbreaks in the United States, 2010–2017

**DOI:** 10.3390/microorganisms9071529

**Published:** 2021-07-17

**Authors:** Danielle M. Tack, Hannah M. Kisselburgh, LaTonia C. Richardson, Aimee Geissler, Patricia M. Griffin, Daniel C. Payne, Brigette L. Gleason

**Affiliations:** Centers for Disease Control and Prevention, Atlanta, GA 30329, USA; Hannah.kisselburgh@cuanschutz.edu (H.M.K.); ijs7@cdc.gov (L.C.R.); ihq5@cdc.gov (A.G.); pmg1@cdc.gov (P.M.G.); dvp6@cdc.gov (D.C.P.); yer7@cdc.gov (B.L.G.)

**Keywords:** Shiga toxin-producing *Escherichia coli* (STEC), O157, non-O157, foodborne, outbreaks, epidemiology

## Abstract

Shiga toxin-producing *Escherichia coli* (STEC) cause illnesses ranging from mild diarrhea to ischemic colitis and hemolytic uremic syndrome (HUS); serogroup O157 is the most common cause. We describe the epidemiology and transmission routes for U.S. STEC outbreaks during 2010–2017. Health departments reported 466 STEC outbreaks affecting 4769 persons; 459 outbreaks had a serogroup identified (330 O157, 124 non-O157, 5 both). Among these, 361 (77%) had a known transmission route: 200 foodborne (44% of O157 outbreaks, 41% of non-O157 outbreaks), 87 person-to-person (16%, 24%), 49 animal contact (11%, 9%), 20 water (4%, 5%), and 5 environmental contamination (2%, 0%). The most common food category implicated was vegetable row crops. The distribution of O157 and non-O157 outbreaks varied by age, sex, and severity. A significantly higher percentage of STEC O157 than non-O157 outbreaks were transmitted by beef (*p* = 0.02). STEC O157 outbreaks also had significantly higher rates of hospitalization and HUS (*p* < 0.001).

## 1. Introduction

An estimated 265,000 Shiga toxin-producing *Escherichia coli* (STEC) infections occur in the United States annually, ranging in severity from mild diarrhea to ischemic colitis and hemolytic uremic syndrome (HUS). Infection can be fatal [1]. STEC are found in the intestinal tract of healthy ruminants (e.g., cattle, goats) and can be transmitted to humans through contaminated food or water, contact with an infected animal or their environment, or directly between persons [2]. 

More than 100 STEC serogroups are associated with human illness; serogroup O157 is the most identified cause of STEC infection in the United States. In 2009, CDC recommended that stool specimens submitted to clinical laboratories from all patients with acute community-acquired diarrhea be tested for Shiga toxin or its genes and simultaneously cultured for *E. coli* O157 [3]. Clinical laboratories have increasingly adopted culture-independent tests to detect Shiga toxin genes, resulting in increased detection of non-O157 STEC [4,5,6,7,8]. In 2010, the incidence of sporadic (not associated with an outbreak) non-O157 STEC infections detected through active sentinel surveillance surpassed STEC O157 infections and has remained higher [6,9].

Most STEC infections are not associated with known outbreaks, but data obtained through outbreak investigations provide important information for understanding modes of transmission and sources. Previous reports have described transmission modes and exposures for O157 and non-O157 STEC outbreaks separately, during different time frames [10,11]. We describe the epidemiology of both O157 and non-O157 STEC outbreaks during 2010–2017 and compare their features. From 2010–2017, STEC O157 outbreaks were reported approximately twice as often as non-O157 STEC outbreaks; however, the distribution of O157 and non-O157 outbreaks varied by age, sex, and severity whereas transmission mode exhibited little variation.

## 2. Materials and Methods

### 2.1. Surveillance

The Centers for Disease Control and Prevention (CDC) conducts surveillance for foodborne, waterborne, and other enteric disease outbreaks through the National Outbreak Reporting System (NORS) web-based platform. Local, state, and territorial health departments report data on outbreaks. We analyzed reports of STEC outbreaks that occurred during 2010–2017 [12]. We examined the primary transmission mode; number of illnesses, hospitalizations, physician-diagnosed HUS, and deaths; patient age and sex distribution; month and year the outbreak began; the state where exposures occurred; the outbreak setting, food preparation setting, type of water exposures, and animal type.

### 2.2. Definitions

We defined an outbreak as ≥2 persons with a laboratory-confirmed STEC infection associated with a common exposure and no other pathogen reported. Laboratory-confirmed cases, as well as epidemiologically linked, clinically compatible cases without laboratory confirmation, were included in case counts. Outbreaks with a serogroup reported were assumed to be culture-confirmed for that serogroup. A serogroup was considered a cause of an outbreak when isolated from ≥2 persons or when isolated from a person and an implicated food or animal. Outbreaks with multiple serogroups were included and categorized as O157, non-O157, or both. Food exposures were categorized according to the Interagency Food Safety Analytics Collaboration (IFSAC) food categorization scheme [13]. Settings were classified as home; childcare; camp; school (includes colleges and universities); other institutions (e.g., hospital, nursing home, shelter, prison/jail); restaurant; farm/dairy; festival/fair; petting zoo; recreational area (e.g., beach, park); and other (e.g., grocery store). States within the contiguous United States were categorized as “north” if north of the 37th parallel and south if below; states with substantial segments crossing this latitude (California and Nevada) were assessed by county; counties with substantial segments crossing the 37th parallel were excluded from geographic analyses.

### 2.3. Analysis

We summarized demographic and epidemiologic findings by transmission mode, STEC subtype, and food category [13]. Comparisons by serogroup were restricted to those caused only by O157 or only by non-O157 serogroups. Geographic analyses included only single state outbreaks. Changes over time were assessed using Sen’s slope. Illness severity was assessed by calculating hospitalization (number hospitalized/total number ill), deaths (number died/total number ill), and HUS (number physician-diagnosed HUS/total number ill). The Kruskal–Wallis test was used to compare median outbreak size and state outbreak rates (outbreaks with exposure in a single state/sum of state populations during 2010–2017, from U.S. Census Bureau intercensal estimates). For categorical analyses we used chi square and Fisher’s exact tests; 2-tailed *p*-values < 0.05 were considered significant. All analyses were performed using SAS v. 9.4 (code available upon request).

## 3. Results

Among the 466 reported STEC outbreaks affecting 4769 persons that occurred during 2010–2017, 459 had a serogroup identified: 330 (71%) were caused by O157, 123 (26%) were caused by non-O157 serogroups, and 6 (1%) included both O157 and non-O157 serogroups (Table 1). The most common non-O157 serogroups were O26 (45, 36%), O111 (24, 20%), and O121 (16, 13%).

**Figure 1:**
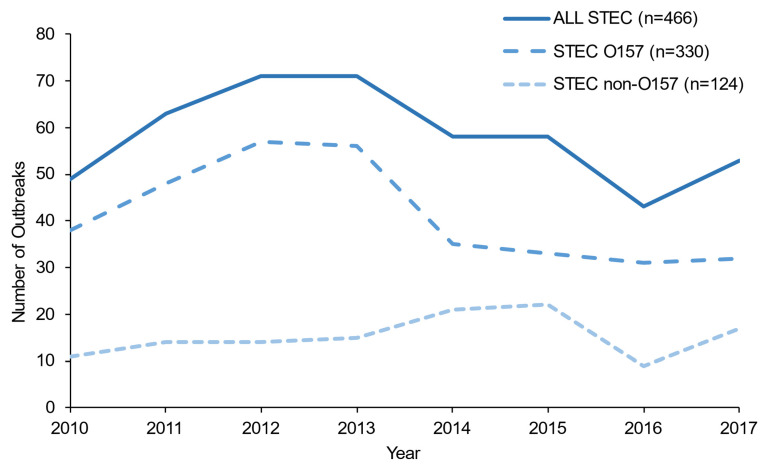
Number of Shiga toxin-producing *Escherichia coli* O157 and non-O157 outbreaks by year, United States, 2010–2017.

There were 3353 illnesses reported for STEC O157 outbreaks and 1047 for non-O157. The median number of illnesses per outbreak was five for both O157 (range 2–105) and non-O157 (range 2–56). STEC O157 caused significantly higher percentages of hospitalization and HUS than non-O157 (*p* < 0.001) (Table 2). The numbers of O157 and non-O157 STEC outbreaks were stable during 2010–2017 (Figure 1).

Transmission modes were foodborne (43%), person-to-person (19%), animal contact (11%), waterborne (4%), environmental contamination (1%), and unknown (23%) (Table 1). There were no significant differences in the percentage of O157 and non-O157 outbreaks by transmission mode: foodborne (44% O157, 41% non-O157; *p* = 0.55), person-to-person (16%, 24%; *p* = 0.08), animal contact (11%, 9%; *p* = 0.53), water (4%, 5%; *p* = 0.67), environmental contamination (2%, 0%; *p* = 0.17), and unknown (23%, 21%; *p* = 0.64) (Table 1). Foodborne disease outbreaks caused the most illnesses (2853, 60%), hospitalizations (776, 67%), HUS cases (137, 45%), and deaths (12, 50%) (Table 2). This was true for O157 outbreaks for illnesses (2017, 60%), hospitalizations (597, 64%), HUS cases (112, 43%), and deaths (11, 50%), and for non-O157 outbreaks for illnesses (567, 54%), hospitalizations (139, 81%), and deaths (1, 50%) (Table 2). 

The median outbreak size differed by transmission mode and food category. Foodborne outbreaks had a significantly greater median size compared with other modes (7.5 vs. 4.0 primary cases, *p* < 0.001); outbreaks with an unknown transmission mode had a significantly lower median outbreak size compared with other modes (3.0 vs. 6.0, *p* < 0.001). Median outbreak size was significantly larger for vegetable row crop outbreaks (15.5 vs. 8, *p* < 0.001) and significantly lower for beef outbreaks (7.5 vs. 10, *p* = 0.018) and dairy outbreaks (5 vs. 11, *p* = 0.005) compared with all other implicated foods. Outbreaks with an unknown food vehicle had a significantly lower outbreak size than outbreaks with an implicated food (3 vs. 6, *p* < 0.001).

### 3.1. Sources 

**Figure 2:**
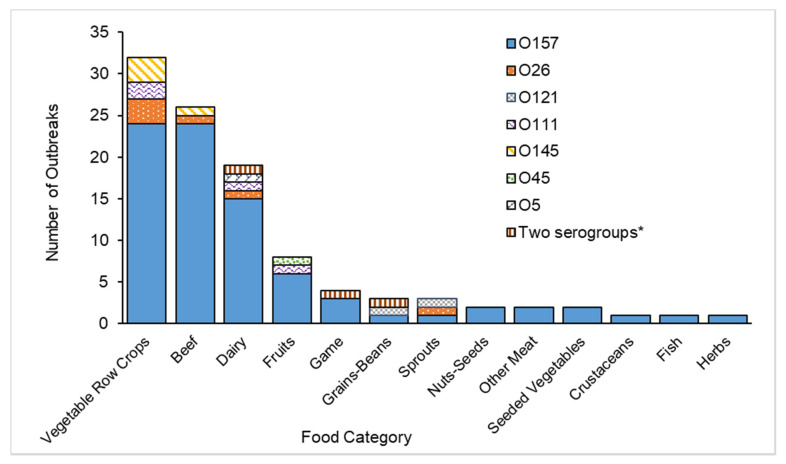
Number of Shiga toxin-producing *Escherichia coli* outbreaks attributed to a single food category (*n* = 104), by serogroup and food category among outbreaks for which this information was determined, United States, 2010–2017. * Two serogroups were isolated from 2 or more persons or foods. Includes one each of O103/O145, O26/O121, and O157/O78.

A food was implicated in 127 (64%) foodborne outbreaks, most commonly vegetable row crops (25%), beef (20%), dairy (15%), and fruit (6%) (Table 1). Thirty of the 32 vegetable row crop outbreaks were associated with leafy greens, most commonly romaine (7 outbreaks) and spinach (5). A higher proportion of foodborne outbreaks of O157 (24/146, 16%) than non-O157 (2/51, 4%) were associated with beef (*p* = 0.02) (Table 1). Most beef outbreaks (22, 85%) were associated with ground beef. All 19 dairy outbreaks were associated with unpasteurized products, usually fluid milk (16, 84%). The non-O157 serogroups that caused the most outbreaks associated with single food categories were O26 (6 outbreaks), O111 (4), and O145 (4) (Figure 2).

Among the 33 outbreaks associated with animal contact, an animal type was implicated in 65% (23) of O157 outbreaks and 82% (9) of non-O157 outbreaks, and a ruminant was an implicated animal type in all but one outbreak (which implicated a pig). Among outbreaks involving a ruminant, 66% involved goats, 63% cattle, and 22% sheep.

Of the 20 waterborne outbreaks, 13 (65%) were O157; of these, 10 were associated with recreational water (7 untreated, 3 treated) and 3 with drinking water. Non-O157 STEC caused 5 untreated and 1 treated recreational water outbreaks.

### 3.2. Severity

Outbreaks were associated with 1163 hospitalizations, 303 cases of HUS, and 24 deaths (Table 2). STEC O157 caused significantly higher rates of hospitalization and HUS than non-O157 STEC (*p* < 0.001) (Table 2). Hospitalization rates differed for outbreaks across transmission modes and food categories. Foodborne (27% vs. 20%, *p* < 0.001) and unknown transmission mode (31% vs. 24%, *p* < 0.001) outbreaks had significantly higher hospitalization rates than the other modes. Dairy outbreaks had a significantly higher hospitalization rate than other outbreaks with identified foods (39% vs. 28%, *p* = 0.007). Compared with other transmission modes combined, animal contact (20% vs. 25%, *p* = 0.008), environmental (15% vs. 25%, *p* = 0.04), and person-to-person (12% vs. 26%, *p* < 0.001) outbreaks had significantly lower hospitalization rates (Table 2). 

HUS rates differed among transmission modes and food categories. Compared with other modes, the rate of HUS was significantly higher for animal contact (9% vs. 6%, *p* = 0.003), waterborne (10% vs, 6%, *p* = 0.042), and unknown transmission mode outbreaks (11% vs. 6%, *p* < 0.001) and significantly lower for foodborne outbreaks (5% vs. 9%, *p* < 0.001). Compared with all other identified foods, the rate of HUS was significantly higher for dairy outbreaks (13% vs. 4%, *p* < 0.001), lower for beef outbreaks (1% vs. 5%, *p* = 0.008), and not significantly different for other identified foods. The death rate was significantly higher for fruit outbreaks compared with all other identified foods (1.9% vs. 0.22%, *p* = 0.038) (Table 2).

STEC O157 outbreaks had significantly higher hospitalization rates than non-O157 STEC outbreaks for person-to-person, foodborne, and unknown transmission modes (Table 2). HUS rates were significantly higher in O157 than non-O157 outbreaks for foodborne, person-to-person, and animal contact transmission modes. No significant differences in death rates were found between O157 and non-O157 outbreaks.

Median hospitalization and HUS rates for O157 and non-O157 outbreaks also differed by food category (Table 2). Among outbreaks associated with dairy, STEC O157 had significantly higher hospitalization rates than non-O157 outbreaks.

### 3.3. Demographic Characteristics

Patient age ranges were available for 4421 (93%) of outbreak-associated illnesses: the highest proportion of illnesses occurred among persons 5–19 (35%). The age group distributions varied by transmission mode. The age group with the highest proportion of persons in person-to-person outbreaks was children < 5 years old (379; 66%). Persons 5–19 years old made up the highest proportion in animal contact (226, 46%) and waterborne outbreaks (116, 67%). For foodborne outbreaks, the age group with the highest proportion was persons 20–49 years old (1039, 40%).

**Figure 3:**
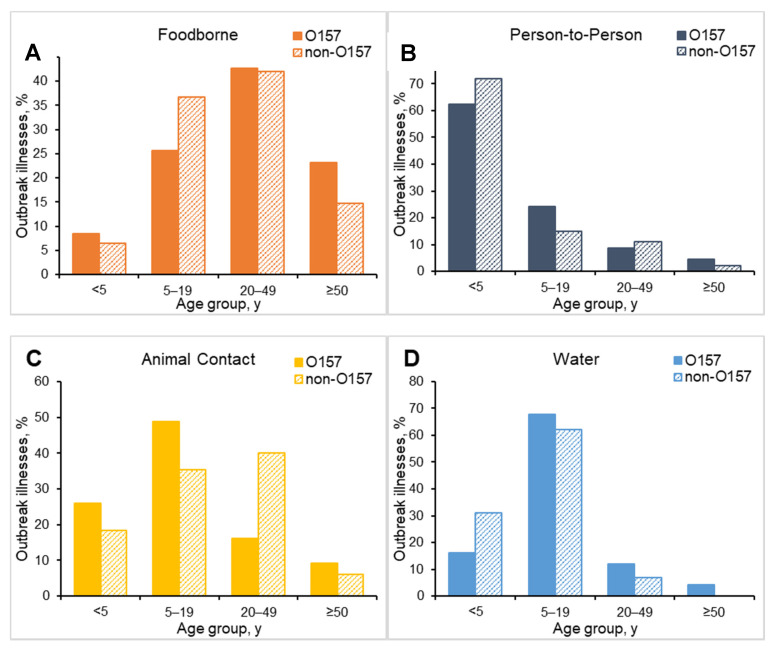
Percentage of Shiga toxin-producing *Escherichia coli* O157 and non-O157 outbreak illnesses by age group and selected transmission modes, United States, 2010–2017. (**A**) foodborne (*n* = 2342); (**B**) person-to-person (*n* = 546); (**C**) animal contact (*n* = 438); (**D**) waterborne (*n* = 172).

**Figure 4:**
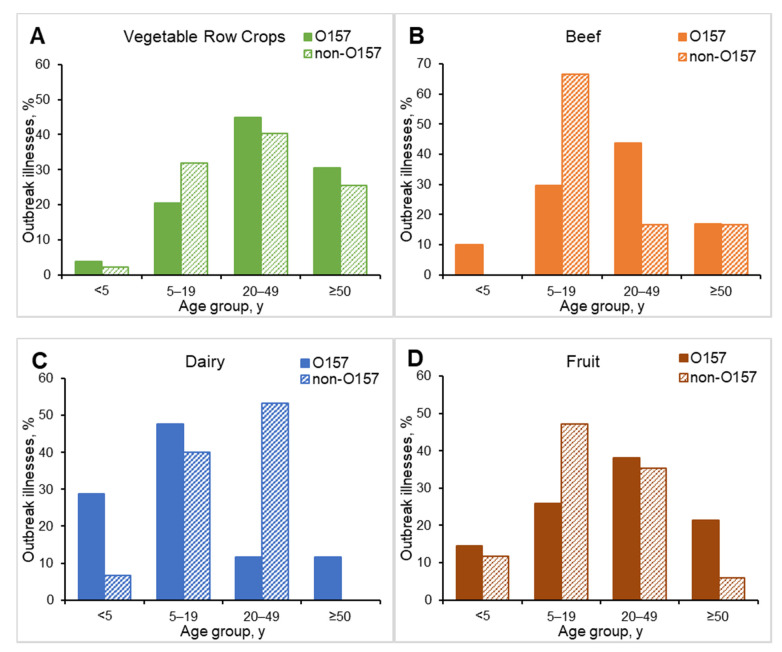
Percentage of Shiga toxin-producing *Escherichia coli* O157 and non-O157 outbreak illnesses by age group and selected food categories, United States, 2010–2017. (**A**) Vegetable row crops (*n* = 738); (**B**) beef (*n* = 211); (**C**) dairy (*n* = 134); (**D**) fruit (*n* = 106).

The proportion of persons in non-O157 STEC outbreaks who were children < 5 years old was higher than in STEC O157 outbreaks (29% vs. 19% O157) and the proportion who were ≥50 years old was lower (9% vs. 17%). The age group distributions among persons in O157 and non-O157 STEC outbreaks varied by transmission modes and food categories (Figure 3 and Figure 4). The largest proportion of illnesses in animal contact outbreaks was among persons 5–19 years old (49%) for STEC O157 but was among persons 20–49 years old (40%) for non-O157 STEC. Although the largest proportion of illnesses in foodborne outbreaks was among persons 20–49 years old and was similar for O157 (43%) and non-O157 (42%), the age distribution for serogroups varied by food category (Figure 4).

More female than male patients were ill in foodborne (51%), environmental (71%), and unknown (54%) transmission mode outbreaks than other modes (*p* < 0.001, *p* = 0.002, and *p* < 0.001, respectively). Slightly more female than male patients were ill in non-O157 (59%) than O157 outbreaks (54%, *p* = 0.011). For foodborne outbreaks, the proportion of females in non-O157 outbreaks was significantly higher than O157 (64% vs. 53%, *p* < 0.001) and for waterborne outbreaks this proportion was significantly lower (35% vs. 60%, *p* = 0.011).

### 3.4. Seasonality and Geography

**Figure 5:**
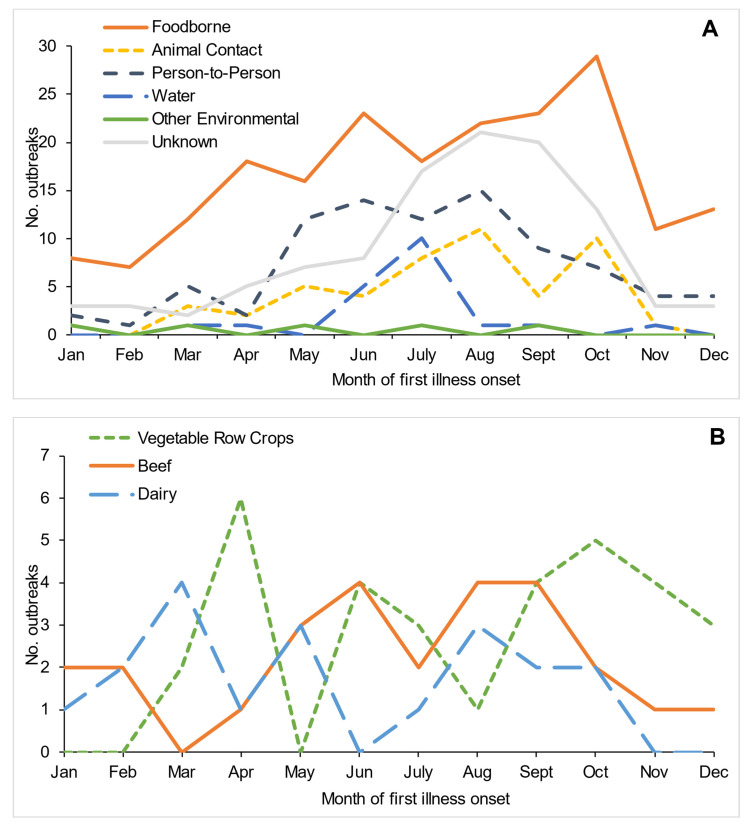
Number of Shiga toxin-producing *Escherichia coli* outbreaks by month, United States, 2010–2017. (**A**) By transmission mode (*n* = 466); (**B**) by selected food categories (*n* = 77).

Two-thirds of STEC outbreaks occurred during the five months from June through October (307, 66%); however, seasonality varied by transmission mode (Figure 5). Foodborne outbreaks occurred year-round with variable seasonality by food category. Peak months for beef outbreaks were May–September (17, 65%); dairy outbreaks peaked in March (4, 21%), May (3, 16%), and July–October (8, 42%), and vegetable row crops outbreaks peaked in April (6, 19%) and October (5,16%). Most person-to-person outbreaks occurred during May–August (53, 61%). Animal contact outbreaks peaked in August (11, 21%) and October (10, 20%). Waterborne outbreaks peaked in July (10, 50%). The seasonal patterns by transmission mode were similar for O157 and non-O157 outbreaks, except non-O157 foodborne outbreaks demonstrated bimodal peaks during April–June (21, 39%) and September–November (14, 27%), and non-O157 animal contact outbreaks had bimodal peaks during June–July (4, 36%) and September–October (4, 36%).

**Figure 6:**
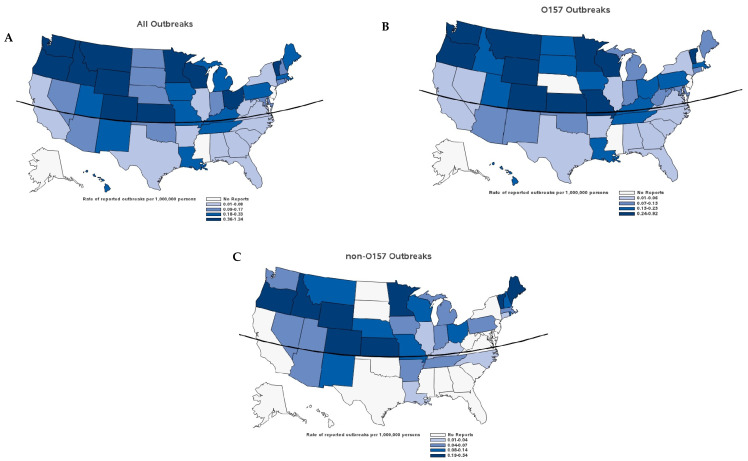
Single-state Shiga toxin-producing *Escherichia coli* outbreaks, by state, United States, 2010–2017. (**A**) All (*n* = 402); (**B**) O157 (*n* = 285); (**C**) non-O157 (*n* = 104). Curved line denotes 37° N latitude.

Single-state outbreaks were reported in 46 states; 43 states reported O157 outbreaks and 33 states reported non-O157 outbreaks (Figure 6). Among the 33 states that reported non-O157 outbreaks, 30 also reported O157 outbreaks. Geographically, the median outbreak rate for STEC was over 2.5 times higher in northern than southern U.S. states (0.20 vs. 0.07 per 1 million persons, *p* = 0.005) (Figure 6). This difference was observed for foodborne and animal contact outbreaks (0.083 vs. 0.032, *p* = 0.04; 0.02 vs. 0.0000, *p* = 0.05), but not for other transmission modes. The higher rate in northern states was true for both O157 (0.16 vs. 0.06, *p* = 0.03) and non-O157 (0.06 vs. 0.00, *p* = 0.004) outbreaks. However, when comparing northern and southern states by transmission mode for O157 and non-O157 the differences identified for all outbreaks were not found.

### 3.5. Settings

A single food preparation setting was reported for 134 (71%) outbreaks (96 O157 outbreaks; 35 non-O157). Food was most often prepared in a restaurant (61, 43%) followed by a private home (35, 26%). The most common food preparation settings were the same for O157 (43% restaurant, 28% home) and non-O157 (48%, 23%) outbreaks.

Among the 82 outbreaks attributed to person-to-person transmission with a reported setting and an identified serogroup, 62 (76%) occurred in a childcare setting (O157 60%; non-O157 37%). A festival or fair was the most common setting for animal contact-associated outbreaks (18, 37%), followed by a petting zoo (12, 24%). Most outbreaks at festivals and petting zoos were O157 (87%). The majority of O157 (54%) and non-O157 (67%) waterborne outbreaks occurred in recreational settings.

## 4. Discussion

During 2010–2017, STEC O157 outbreaks and illnesses were reported approximately three times as often as non-O157 STEC outbreaks and illnesses. STEC O157 outbreaks resulted in more severe illnesses. Food was the principal mode of transmission for outbreaks caused by both O157 and non-O157 serogroups [10,11]. 

More foodborne STEC outbreaks were associated with vegetable row crops than any other food category. Although vegetable row crops and beef contributed relatively similarly to O157 outbreaks, vegetable row crops were responsible for significantly more non-O157 outbreaks than beef. STEC O157 was declared an adulterant in ground beef in 1996, followed by the six non-O157 serogroups in 2011. Increased regulation of produce occurred in 2016 with the implementation of the Produce Safety Rule [14]. This regulation establishes minimum standards for growing, harvesting, packing, and holding fruits and vegetables meant for human consumption. Outbreaks associated with romaine lettuce in 2018 led to changes in agricultural marketing agreement guidelines intended to decrease STEC contamination in fields [15,16]. The implementation of practices in these regulations and guidelines could decrease the number of outbreaks associated with vegetable row crops and other produce.

The proportion of outbreaks associated with various food types for O157 and non-O157 were similar except for beef. It is not known why beef was the only category with significantly more O157 than non-O157 outbreaks given that non-O157 STEC are more commonly detected on beef carcasses [17]. One possibility is that detection of outbreaks might be correlated with production of Shiga toxin 2 (most STEC O157 produce only Shiga toxin 2); strains that produce only Shiga toxin 2 cause the most severe illnesses, followed by those that produce both Shiga toxins 1 and 2, then only Shiga toxin 1 [18,19,20]. Shiga toxin types were not available for these outbreaks, but Shiga toxin 1 is more common in isolates of bovine origin [18]. It is also possible that some mixed infections might be reported as O157 when additional testing to identify another serogroup was not attempted after O157 was identified, thus underestimating the contribution of non-O157 strains in outbreaks. 

STEC O103 is the second most commonly reported non-O157 STEC causing human illness [21] and the serogroup most commonly isolated from cattle [18,22,23]. However, it caused only 3% of STEC outbreaks in our analysis. This could be because about 95% of O103 isolates from U.S. residents produce only Shiga toxin 1 [24,25].

Foodborne outbreaks were larger than those caused by other transmission modes. The wide distribution of food might explain this difference. Contaminated food can cross state borders, resulting in many cases occurring over a short period before the outbreak can be detected, due to intrinsic lags in surveillance systems. These cases can prompt rigorous investigations in multiple jurisdictions, resulting in many confirmed, epidemiologically-linked cases. Whereas beef is also widely distributed, the larger size of vegetable row crop outbreaks could be the result of these foods often being consumed raw, unlike beef. Therefore, more people may be exposed to STEC by consuming vegetables raw. Like beef and vegetable row crops, dairy can be distributed across multiple states. However, most dairy outbreaks were from unpasteurized milk which is prohibited by federal law from being distributed across state lines, thereby limiting exposures.

The variation in the age and sex distribution of STEC outbreaks overall and by serogroup category is likely a reflection of general differences in behavior (health seeking, hand washing, recreation, etc.) and food choices. For example, more person-to-person outbreaks occur among young children than adults and more STEC O157 dairy outbreaks occur among children than adults. These findings are consistent with previous reports [10,11].

Consistent with disease surveillance of sporadic infections and previous studies, STEC O157 outbreaks resulted in more hospitalizations and HUS than non-O157 [4,26]. This difference is likely due to the toxin profile; unlike most non-O157 STEC, most STEC O157 strains isolated from U.S. residents produce only Shiga toxin 2. Strains that produce only Shiga toxin 2 cause more severe illness than those that produce both Shiga toxins 1 and 2 or only Shiga toxin 1 [4,18,19,20,26]. Differences in toxin profile could also influence the detection of the source of an outbreak, as persons infected with less virulent strains may differ in medical care-seeking behaviors, therefore affecting the detection and laboratory confirmation of that serogroup and source. Outbreaks of more severe illnesses might also be more rigorously investigated by public health officials.

Higher hospitalization and HUS rates attributed to different transmission modes could also be influenced by age. For example, foodborne outbreaks were associated with significantly more hospitalizations, and the foodborne transmission mode was the most common mode among persons ≥ 50 years old. However, animal contact and waterborne outbreaks had significantly higher rates of HUS in persons < 20 years and most of these outbreaks occurred in persons < 20 years [27]. As has been noted before, person-to-person outbreaks did not have significantly higher HUS rates despite most person-to-person outbreaks occurring in children < 5 years [10]. This might be explained by a large proportion of illnesses in person-to-person outbreaks being associated with non-O157 serogroups, which had significantly lower rates of hospitalization and HUS than O157.

Our analyses identified seasonal patterns by transmission mode consistent with those previously reported [10,28]. We found that increases in outbreaks during April and October appear to correspond with peaks in vegetable row crop outbreaks and mark the start of leafy green (a subcategory of vegetable row crops) harvest in different production areas [29]. Additional information on the ecology of the growing regions and variations in farming practices is needed to further assess this observation.

The incidence of STEC O157 infection is higher in northern than southern regions of the United States [10,30]. We found that northern regions had more O157 and non-O157 STEC outbreaks. We found similar geographic differences for foodborne and animal contact outbreaks. These findings suggest that the distribution of STEC is likely related to ecologic differences. However, differences in cattle production systems, the proportions of people exposed to particular foods or environments, eating habits, and reporting biases might have an influence. 

STEC outbreak surveillance outside the United States is primarily focused on foodborne outbreaks. Pires, et al. used outbreak data from 27 countries across three regions during 1998–2017 to attribute STEC infections to various food categories. In their model, the top food categories in the American and European regions were beef and produce, whereas the top categories in the Western Pacific region were produce and dairy [31]. Our findings were similar in that vegetable row crops, beef, dairy, and fruit were the most common food categories identified as sources of outbreaks and outbreak-associated illnesses. However, our analysis, which encompasses the last 8 years of the Pires, et al. analysis, indicates that produce (vegetable row crops and fruits) caused more STEC outbreaks and outbreak-associated illnesses than beef.

This analysis has several limitations. The data we assessed are limited to investigated and reported outbreaks and may not be representative of all STEC outbreaks. Information on hospitalization, HUS, and death was not available for all outbreak-associated cases, so rates may not accurately reflect the true rates for these severity indicators. Differences identified for sex and age groups might be due to differences in the availability of sex or age information. Smaller outbreaks, many of which had an undetermined source, had less evidence to help determine the source or transmission mode. The ability to identify statistically significant differences between O157 and non-O157 outbreaks for specific sub-analyses (e.g., geographical differences based on transmission mode) was limited by the small numbers of outbreaks within some categories. Finally, the database is dynamic, and data may be updated at any time, even years after an outbreak, which could affect subsequent analyses. 

Most STEC outbreaks in the United States continue to be attributed to foodborne transmission. Outbreaks are dominated by the O157 serogroup despite higher detection of non-O157 serogroups among sporadic infections. STEC O157 outbreaks continue to cause more severe disease than non-O157 STEC. The recent adoption of whole genome sequencing might improve the identification and investigation of STEC outbreaks; this could provide more detailed information on sources and virulence factors. Our analyses found both similarities and significant differences based on the mode of transmission by food category, age, sex, and setting which can be used to target interventions to groups at higher risk. Continued STEC outbreak surveillance is needed to identify changes in epidemiology and to assess the effect of prevention efforts. 

## Figures and Tables

**Table 1:** Number of Shiga toxin-producing *Escherichia coli* (STEC) outbreaks, by primary transmission mode and food category, United States, 2010–2017 *.

	STEC		Serogroup Category					Top non-O157 Serogroups					
	Total †		O157		Non-O157 ‡		*p*	O26		O111		O121	
Transmission source	No.	(%)	No.	(%)	No.	(%)		No.	(%)	No.	(%)	No.	(%)
Food	200	(43)	146	(44)	51	(41)	0.55	18	(40)	7	(30)	8	(50)
Veg. row crops	32	(16)	24	(16)	8	(16)	0.90	3	(16)	2	(29)	0	(0)
Beef	26	(13)	24	(16)	2	(4)	**0.02**	1	(6)	0	(0)	0	(0)
Dairy	19	(10)	15	(10)	3	(6)	0.35	1	(6)	1	(14)	0	(0)
Fruit	8	(4)	6	(4)	2	(4)	0.95	0	(0)	1	(14)	0	(0)
Other foods §	42	(21)	29	(20)	12	(24)	0.58	1	(6)	1	(14)	5	(63)
Unknown	73	(37)	48	(32)	24	(47)	0.07	12	(66)	2	(29)	3	(37)
Person-to-person	87	(19)	54	(16)	29	(24)	0.08	14	(30)	8	(33)	3	(19)
Animal contact	49	(11)	36	(11)	11	(9)	0.53	4	(9)	3	(13)	0	(0)
Water	20	(4)	13	(4)	6	(5)	0.67	0	(0)	2	(8)	2	(13)
Other environmental	5	(1)	5	(2)	0	(0)	0.33 ¶	0	(0)	0	(0)	0	(0)
Unknown transmission	105	(23)	76	(23)	26	(21)	0.64	9	(20)	4	(17)	3	(19)
TOTAL	466	(100)	330	(71)	123	(26)	NA	45	(36)	24	(20)	16	(13)

* Bold font indicates statistical significance at *p* < 0.05; † Includes 6 outbreaks caused by both O157 and non-O157 (3 food: 1 dairy, 1 other foods, 1 food unknown; 1 person-to-person; 1 animal contact; 1 unknown) and 7 outbreaks of unknown serogroup (3 person-to-person, 1 animal contact, 1 water, 2 unknown); ‡ Includes the following serogroups O26 (45 outbreaks), O111 (24), O121 (16), O103 (12), O145 (9), O45 (7), O5 (3), multiple (3), O71 (1), O118 (1), O186 (1), and unknown (1); § Other foods include game (4 outbreaks), grains-beans (3), sprouts (3), seeded vegetables (2), nuts-seeds (2), other meat (2), herbs (1), fish (1), crustaceans (1), and outbreaks not attributed to a single food category (23). Outbreaks not attributed to a single food category could not be assigned to one of the 24 aggregate food categories, which includes outbreaks due to complex foods for which the contaminated ingredient was not determined (e.g., pico de gallo, sandwich, sausage), those with ill-defined implicated foods (e.g., buffet, appetizer), and those due to more than one food category; ¶ Fisher’s exact.

**Table 2:** Characteristics of Shiga toxin-producing *Escherichia coli* outbreaks, by primary transmission mode and food category, United States, 2010–2017.

Transmission Mode and Source	Illnesses No. (Median Size)						Hospitalizations No. (%)						HUS * No. (%)						Deaths No. (%)					
	All STEC †		O157		Non-O157		All STEC †		O157		Non-O157		All STEC †		O157		Non-O157		ALL STEC †		O157		Non-O157	
Food	2853	(7.5)	2017	(8)	567	(6)	776	(27) §	597	(30) #	139	(25)	137	(5) ¶	112	(6) **	7	(1)	12	(0.4)	11	(0.5)	1	(0.2)
Veg. row crops	738	(15.5)	613	(15)	125	(16)	223	(30)	183	(30)	40	(32)	31	(4)	27	(4)	4	(3)	2	(0.3)	2	(0.3)	0	(0)
Beef	211	(7.5)	200	(8)	11	(6)	60	(28)	57	(29)	3	(27)	2	(1) ¶	2	(1)	0	(0)	1	(0.5)	1	(0.5)	0	(0)
Dairy	148	(5)	119	(4)	15	(6)	57	(39) §	49	(41) #	1	(7)	19	(13) §	16	(13)	0	(0)	0	NA	0	(0)	0	(0)
Fruit	106	(6.5)	89	(7)	17	(9)	24	(23)	23	(26)	1	(6)	3	(3)	3	(3)	0	(0)	2	(1.9) §	2	(2.2)	0	(0)
Other foods ‡	738	(10.5)	507	(12)	220	(13)	195	(26)	144	(28)	47	(21)	33	(4)	30	(6) #	3	(1)	1	(0.1)	0	(0)	1	(0.5)
Unknown	912	(4)	489	(4)	179	(4)	217	(24)	141	(29)	47	(26)	49	(5)	34	(7) **	0	(0)	6	(0.4)	6	(1)	0	(0)
Person-to-person	587	(5)	293	(4)	269	(6)	70	(12) ¶	68	(23) **	1	(0.4)	36	(6)	36	(12) **	0	(0)	3	(0.5)	3	(1)	0	(0)
Animal contact	509	(4)	387	(4)	68	(4)	100	(20) ¶	81	(21)	12	(18)	48	(9) §	44	(11) #	1	(2)	3	(0.6)	3	(0.8)	0	(0)
Water	218	(6.5)	181	(8)	31	(5)	46	(21)	42	(23)	4	(13)	21	(10) §	19	(11)	2	(7)	1	(0.5)	1	(0.6)	0	(0)
Other environmental	86	(6)	86	(6)	0	NA	13	(15) ¶	13	(15)	NA		5	(6)	5	(6)	NA		0	(0)	0	(0)	NA	
Unknown transmission	516	(3)	389	(3)	112	(4)	158	(31) §	138	(36) **	16	(14)	56	(11) §	46	(12)	8	(7)	5	(1)	4	(1)	1	(0.9)
TOTAL	4769	(5)	3353	(5)	1047	(5)	1163	(24)	939	(28)**	172	(16)	303	(6)	262	(8) **	18	(2)	24	(0.5)	22	(0.7)	2	(0.2)

* HUS, hemolytic uremic syndrome;†Includes 6 outbreaks caused by both O157 and non-O157 (3 food: 1 dairy, 1 other foods, 1 food unknown; 1 person-to-person; 1 animal contact; 1 unknown) and 7 outbreaks of unknown serogroup (3 person-to-person, 1 animal contact, 1 water, 2 unknown); ‡ Other foods include game (4 outbreaks), grains-beans (3), sprouts (3), seeded vegetables (2), nuts-seeds (2), other meat (2), herbs (1), fish (1), crustaceans (1), and outbreaks not attributed to a single food category (23). Outbreaks not attributed to a single food category could not be assigned to one of the 24 aggregate food categories, which includes outbreaks due to complex foods for which the contaminated ingredient was not determined (e.g., pico de gallo, sandwich, sausage), those with ill-defined implicated foods (e.g., buffet, appetizer), and those due to more than one food category; § *p* < 0.05; higher than the comparison group, which was all transmission modes combined or all other assigned food categories combined; ¶ *p* < 0.05; lower than the comparison group, which was all transmission modes combined or all other assigned food categories combined; # *p* < 0.05; ** *p* < 0.001.

## Data Availability

The data presented in this study are available on request from the corresponding author. Due to PII and propriety information, a complete NORS dataset is not publicly available. A subset of key variables from the NORS database is publicly available at https://wwwn.cdc.gov/norsdashboard/ and the full dataset can be requested through NORSdashboard@cdc.gov.
